# Anatomical Feasibility Study on Novel Ascending Aortic Endograft With More Proximal Landing Zone for Treatment of Type A Aortic Dissection

**DOI:** 10.3389/fcvm.2022.843551

**Published:** 2022-04-06

**Authors:** Xiaoye Li, Longtu Zhu, Lei Zhang, Chao Song, Hao Zhang, Shibo Xia, Wenying Guo, Zaiping Jing, Qingsheng Lu

**Affiliations:** Division of Vascular Surgery, Department of General Surgery, Changhai Hospital, Naval Medical University, Shanghai, China

**Keywords:** endovascular repair, type A aortic dissection (TAAD), anatomical feasibility study, novel endograft, aortic dissection (AD)

## Abstract

**Objective:**

Type A aortic dissection (TAAD) is associated with high morbidity and mortality, and open surgery is the best treatment option. Development of endovascular repair devices for TAAD will benefit patients deemed unfit for open surgery. In this study, we performed a thorough investigation of anatomical features in Asian patients with TAAD to learn about the patient eligibility of a novel ascending aortic endograft technique.

**Methods:**

Computed tomography angiography (CTA) images of TAAD cases in our institution from January 2015 to November 2021 were reviewed, and three-dimensional reconstructions were performed with the Endosize software (Therenva, Rennes, France). Anatomic structures including length measured along centerline and greater/lesser curvature, ascending aorta/aortic root dimensions, as well as location of entry tear and extent of dissection were analyzed.

**Results:**

A total of 158 patients were included [median age 58 years, interquartile range (IQR), 30–76 years; 115 males, 72.8%]. In 99 (62.7%) of the cases, entry tear was distal to the sinotubular junction (STJ). In 106 (67.1%) of the cases, the pathology proximally extended into the aortic root, which was intramural hematoma in 37 (23.4%) of the cases, and the aortic root was free from the pathology in 52 (32.9%) of the cases. The median distance from the STJ to the proximal edge of the ostium of the innominate artery (IA) measured along the centerline was 65 mm (IQR 58–74 mm). The median distance from the distal edge of the higher coronary ostium to the STJ was 7.95 mm (IQR 5.625–10.9 mm). The bare metal stent part was set between the edge of the higher coronary ostium and the STJ. In our series, 63 (39.9%) of the cases had this distance >10 mm. The relative difference was <20% between the STJ and the proximal edge of the ostium of the IA in 92 (58.2%) of the cases. Ascending aorta radius of curvature was 52.2 mm (IQR 43.7–63.7 mm).

**Conclusions:**

Our study demonstrates that 56.3% of the TAAD cases would be amenable to endovascular repair by the novel ascending aortic endograft, with sufficient landing zone free of the dissected aorta.

## Introduction

Stanford type A aortic dissection (TAAD) is one of the most catastrophic emergent aortic diseases; the mortality rate of which could be up to 57% with medical treatment (1, 2). Surgery is still the only viable treatment option for TAAD. Development of cardiopulmonary bypass, anesthetic techniques, and surgery techniques have greatly improved perioperative outcomes, with in-hospital mortality rate decreasing from 25 to 18%. About 16.3 to 50% TAAD patients were deemed inoperable because of advanced age, severe comorbidities and hemodynamic instability, and some patients were reluctant to surgery. Consequently, endovascular repair for TAAD is of great potential application and has attracted clinicians from all over the world.

In the last decade, many clinicians reported the outcomes of endovascular repair for TAAD, most of which were case reports and case series (3). Off-label use of thoracic endograft was most common in these studies. Compared with the thoracic aorta, the ascending aorta exhibits a more significant pulsatile change in diameter, and endograft would cause greater pressure in each cardiac cycle (4). A healthy proximal landing zone is rare in patients with TAAD, since the pathology would extend to the lower 1/3 of the ascending aorta or even the aortic root in most cases (5). Besides, the ascending aorta is more curved, requiring the endograft to have better flexibility to avoid inadequate sealing, which would result in bird-beak configuration and type I endoleak.

Considering the significant difference in morphological and physiological characteristics between the ascending aorta and the descending aorta, a dedicated ascending aortic endograft technique is desperately needed for endovascular repair of TAAD. In this study, we aim to evaluate the feasibility of extending endograft into the region between the distal edge of the higher coronary ostium and the sinotubular junction (STJ) in patients with TAAD, and collect data for future design and manufacture of a dedicated ascending aortic endograft device.

## Materials and Methods

### Patient Population

Between January 2015 and November 2021, a total of 1,060 patients were discharged from our center with a diagnosis of “aortic dissection.” The report on the computed tomography angiography (CTA) of each patient was studied, and those without CTA examination or diagnosed with type B aortic dissections were excluded. In total, 164 patients diagnosed with type A aortic dissections by available preoperative high-resolution CTA were included, but 6 patients were excluded because of previous artificial aortic valve implantation. The institutional review committee approved this retrospective study, and the need for informed consent was waived.

### Imaging Analysis

Aortic diameters and segmental lengths were analyzed according to CTA. An axial slice thickness of 1 mm or less was present in all the patients. All CTA image sets were analyzed with the Endosize software (Therenva, Rennes, France) for three-dimensional spatial analysis. All measurements were taken by multiplanar reconstruction always in the plane perpendicular to the manually corrected local aortic centerline. Entry tear location was determined by discontinuity in the intima with visualization of contrast flow into the false lumen. The plane of the aortic annulus was defined by the virtual basal ring, or the lowest point of eachaortic valve leaflet's attachment point to the sinus of Valsalva. This plane also served as the reference point along the centerline. The STJ was signified by transition from the bulbous portion of the sinuses of Valsalva to the tubular proximal ascending aorta.

The aortic length L1-L3 is measured along the centerline from the distal edge of the aortic annulus to the: (1) distal edge of the left coronary ostium (L1); (2) distal edge of the right coronary ostium (L2); and (3) STJ (L3). Length from the distal edge of the left coronary ostium to the STJ (L4) was calculated by subtraction: L4 = L3–L1. The same is applied for the length from the distal edge of the right coronary ostium to the STJ (L5): L5 = L3–L2. The aortic length L6-L8 is measured along the center line from the STJ to the: (1) proximal extent of the dissected aorta or intramural hematoma (L6); (2) primary entry tear (L7); and (3) proximal edge of the ostium of the innominate artery (IA) (L8). For L6 and L7, if the proximal extent of pathology and primary entry tear was distal to the STJ, the distance would be documented as “proximal to the STJ.” The aortic length from the STJ to the proximal edge of the ostium of the IA was also measured on the greater curvature side (L9) and the lesser curvature side (L10). In cases where the STJ was not definable, it was set 10 mm distally to the origin of the more distal coronary artery.

According to L8, the ascending aorta was divided into 4 equal parts. The diameter of the following was measured co-axially because of the centerline flow axis: (1) sinus of the Valsalva (D1); (2) STJ (D2); (3) 1/4 plane of the ascending aorta (D3); (4) 1/2 plane of the ascending aorta (D4); (5) 3/4 plane of the ascending aorta (D5); (6) plane at the proximal edge of the ostium of the IA (D6). (D2-D5)/D2 is calculated to determine whether tapered stent-graft is required.

**Figure 1:**
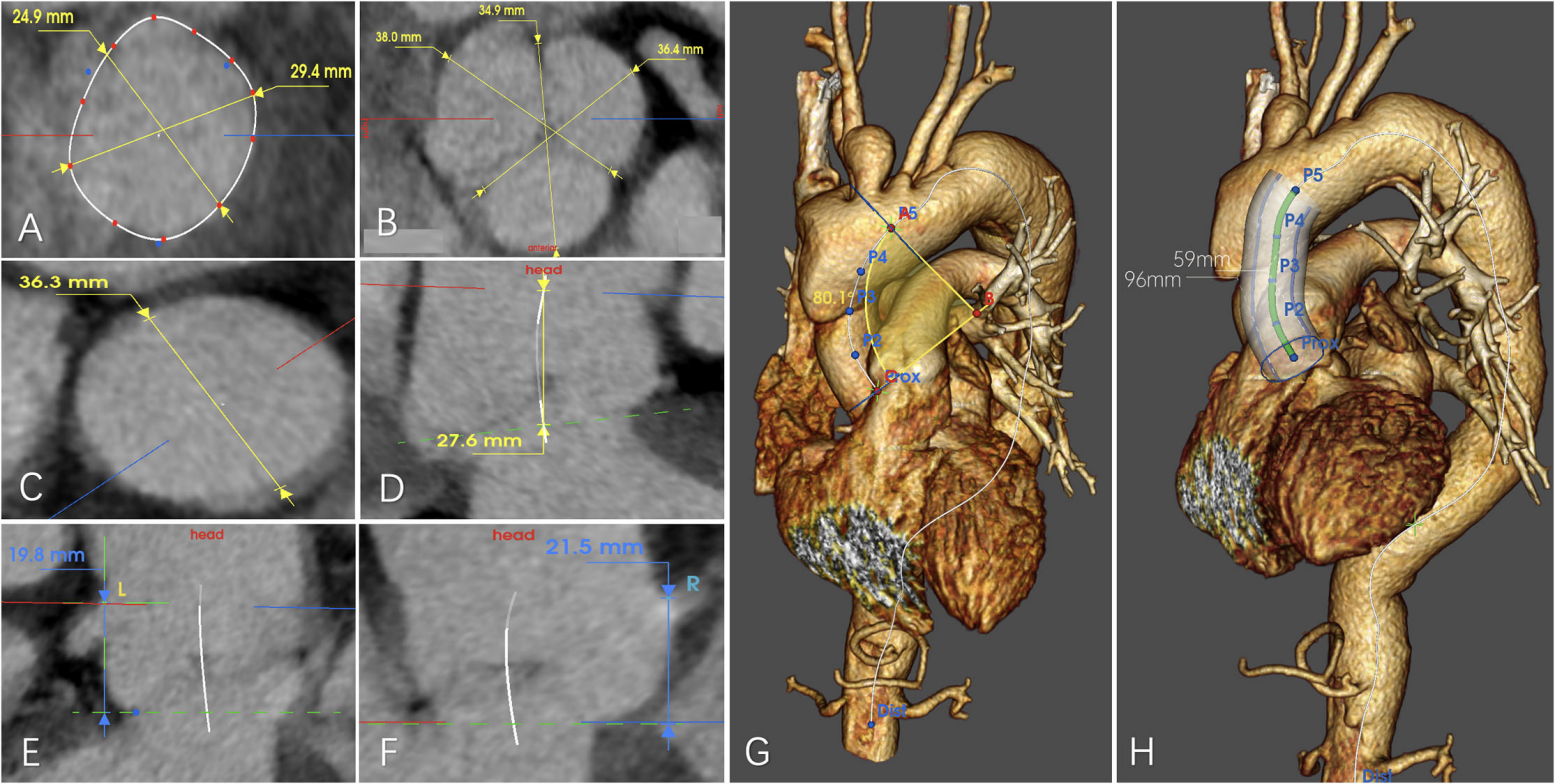
Example for anatomical measurement of **(A)** diameter of the aortic annulus, **(B)** diameter of the sinus of the Valsalva, **(C)** diameter of the sinotubular junction (STJ), **(D)** distance between the aortic annulus and the STJ, **(E)** Distance between the aortic annulus and the distal edge of the left coronary ostium, **(F)** distance between the aortic annulus and the distal edge of the right coronary ostium, **(G)** aortic radius of curvature, and **(H)** length between the STJ and the distal edge of the ostium of the innominate artery measured along the centerline/greater curvature side/lesser curvature side.

The ascending aorta radius of curvature was calculated to assess the tortuosity of the ascending aorta, based on the angle between the STJ and the ostium of IA centerlines, and the length of the ascending aorta. The angle was composed of the line perpendicular to the centerline of the STJ, the line perpendicular to the centerline of the proximal ostium of the IA, the intersection of the line perpendicular to the centerline of the STJ, and the line perpendicular to the centerline of the proximal ostium of the IA. Ascending aorta radius of curvature = (180^*^length of ascending aorta)/(angle^*^π) (Figure 1).

### Device Requirements

The simulated tubular stent-graft is composed of two parts: a bare metal stent part (proximal 10mm) and a covered stent part (the rest part). The bare metal stent is intended to protrude into the aortic root, the junction of the bare metal stent and the covered stent is right in the position of the STJ, and the covered stent is in the ascending aorta. The bare metal stent is structured for fixation, and located in the segment between the distal edge of the higher coronary ostium and the STJ. The distal landing zone is defined as 10 mm proximal to the ostium of the IA for simple interpretation of outcomes, because this region is mostly involved in the pathology and, thus, not an ideal landing zone in conventional thoracic endovascular repair (TEVAR). Inclusion criteria were: (1) distance between the distal edge of the higher coronary ostium and the STJ > 10 mm; (2) entry tear located distally to the STJ; (3) aortic root free from the pathology. When the aortic root is proximally involved by intramural hematoma, it was deemed as a relative indication and calculated exclusively. Exclusion criterion was entry tear in the aortic root or aortic root is proximally involved by the dissected aorta.

### Statistical Analysis

All values are expressed as median (first quartile, third quartile) or as number (percentage). Measurement was performed by two independent clinicians, with <5% variation in reported values considered as acceptable. If a ≥5% variation occurred, measurement would be performed by another more experienced clinician.

## Results

All the patients were Asian and had a median age of 58 years (IQR 30–76 years), including 115 (72.8%) males. In 50 (31.6%) of the cases, the primary entry tear was found in a coverable zone in the ascending aorta, and in the distal landing zone in 16 (10.1%) of the cases. In 33 (20.9%) of the case, the primary entry tear was found beyond the distal landing zone. In 25 (15.8%) of the cases, the primary entry tear was unidentified. Considering only the location of entry, endovascular repair with the device would be possible in 99 (62.7%) of the cases.

**Figure 2:**
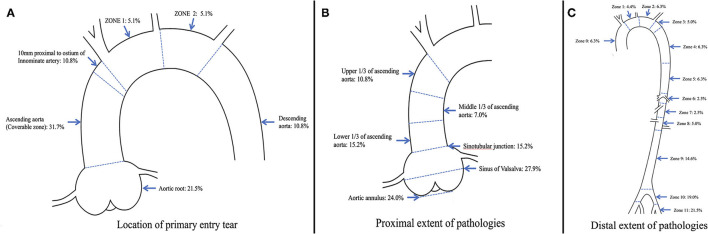
Distribution of **(A)** location of primary entry tear, **(B)** proximal extent of pathologies, and **(C)** distal extent of pathologies in 158 patients with type A aortic dissection.

In 106 (67.1%) of the cases, the pathology proximally extended into the aortic root, which was intramural hematoma in 37 (23.4%) cases. In 52 (32.9%) of the cases, the aortic root was free from the pathology, including proximally extended to the upper 1/3 of the ascending aorta in 17 cases, middle 1/3 of the ascending aorta in 11 cases, and lower 1/3 of the ascending aorta in 24 cases. Among these patients, the pathology was entirely confined in the ascending aorta in 5 (3.2%) of the cases. In total, 89 (56.3%) of the cases met the relative indication. For the supra-aortic trunks, the innominate artery (IA) was involved in the pathology in 64 (40.5%) of the cases, the left common carotid artery (LCCA) was involved in 43 (27.2%) of the cases, and the left subclavian artery (LSA) was involved in 42 (26.6%) of the cases. The pathology distally extending into zone 9 or beyond was most common, and accounted for 55.1% (*n* = 87) of all the cases. Figure 2 provides an overview of the distribution of the primary entry tear and proximal and distal extent of the pathology.

**Figure 3:**
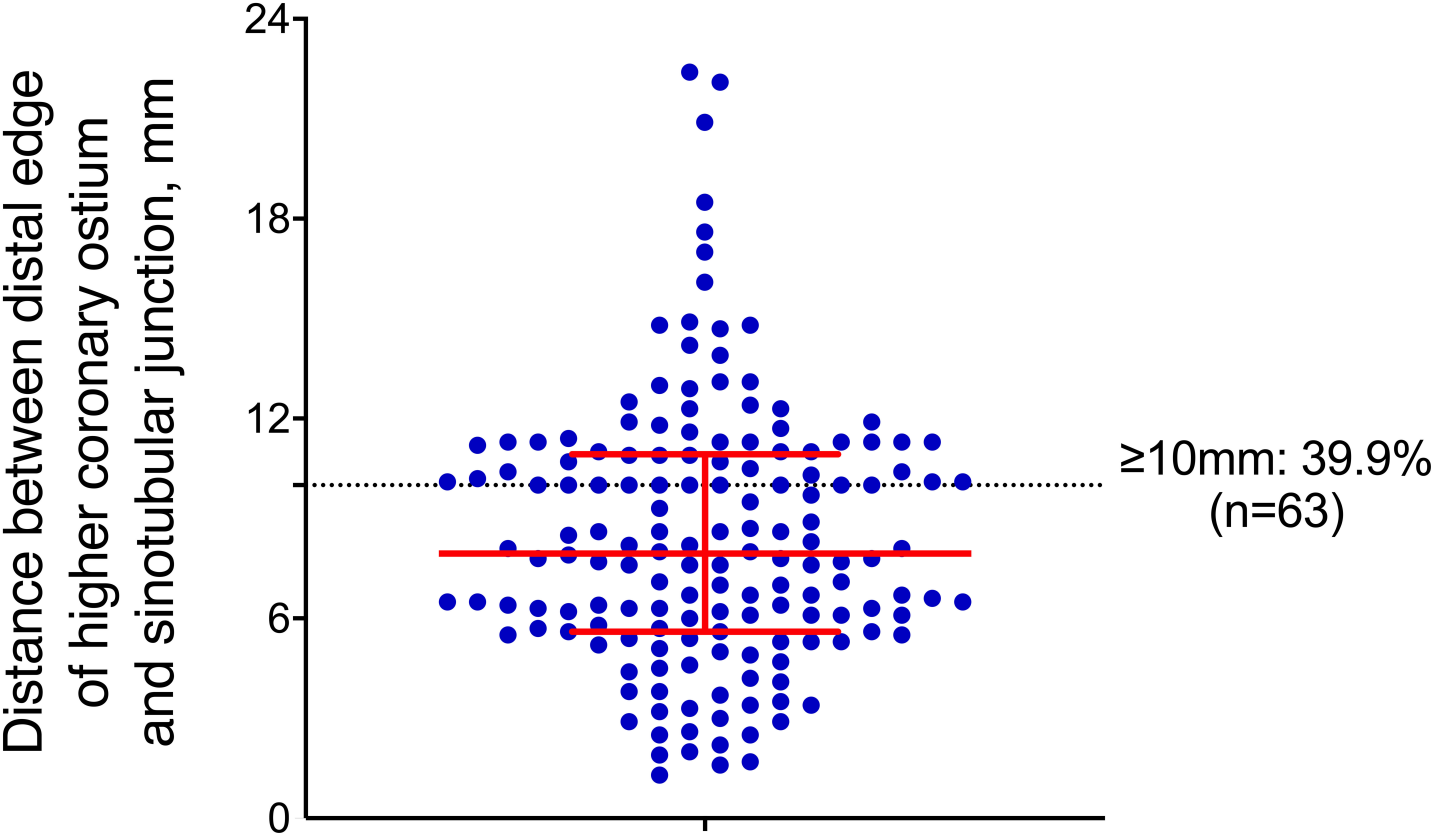
Distance between the distal edge of the higher coronary ostium and STJ, where the red bar indicates the median, 1st-3rd interquartile ranges, and each blue dot represents a single patient.

Measurements of the ascending aorta and aortic root are summarized in Table 1. The left coronary ostium was higher in 83 (52.5%) of the cases. The median distance from the distal edge of the higher coronary ostium to the STJ was 7.95 mm (IQR 5.625–10.9 mm). The median distance from the STJ to the proximal edge of the ostium of the IA measured along the centerline was 65 mm (IQR 58–74 mm), and 154 (97.5%) of the cases can be categorized into 7 different length intervals of 10 mm, ranging from 40 to 110 mm. The distance was <40 mm in 4 of the cases (2.5%; 17, 19, 29, and 38 mm). In 106 (67.1%) of the cases, the distance between the STJ and the proximal extent of the pathology was documented as “proximal to the STJ,” while in the remaining 52 (32.9%) cases, the median distance between the STJ and the proximal extent of the pathology was 15 mm (IQR 4–49 mm). The bare metal stent part was set between the edge of the higher coronary ostium and the STJ. In our series, 3 (1.9%) of the cases had a distance ≥20 mm, 7 (4.4%) had a distance ≥15 mm, 63 (39.9%) had a distance ≥10 mm, and 134 (84.8%) had a distance ≥5 mm (Figure 3).

**Table 1:** Anatomical measurements [median (interquartile range), *n* = 158].

**Diameter, mm**	
Aortic annulus	28.0 (25.7–32.5)
Sinus of Valsalva	38.3 (35.8–44.4)
STJ	32.3 (29.4–37.7)
1/4 ascending aorta	34.5 (29.5–41.4)
1/2 ascending aorta	33.6 (27.8–41.1)
3/4 ascending aorta	32.5 (26.9–38.2)
Proximal edge of ostium of IA	33.8 (30.8–37.6)
**Length, mm**	
Aortic annulus-STJ	28 (23.2–30.6)
Distal edge of left coronary ostium-STJ	10.1 (7.0–13.0)
Distal edge of right coronary ostium-STJ	10 (7.0–14.1)
Distal edge of higher coronary ostium-STJ	8.0 (5.6–10.9)
STJ-Proximal extent of pathology	15.1 (4.0–48.8)
STJ-Primary entry tear	39.9 (15.3–82.2)
STJ- Proximal edge of ostium of IA (centerline)	65.0 (58.0–74.0)
STJ- Proximal edge of ostium of IA (greater curvature side)	84.0 (76.3–98.8)
STJ- Proximal edge of ostium of IA (lesser curvature side)	47.5 (40.3–55.0)
Angle between the centerline of STJ and proximal edge of ostium of IA, degrees	73.5 (59.7–87.7)
Ascending aortic radius of curvature, mm	52.2 (43.7–63.7)

*STJ, sinotubular junction; IA, innominate artery*.

Diameters were measured along the centerline of the true lumen. In 19 (12%) of the cases, the diameter of the STJ was longer than 40 mm. The median absolute difference between the STJ and the proximal edge of the ostium of the IA was 5.2 mm (IQR 2.125–9.35 mm). The relative difference was <10, 15, and 20% between the STJ and the proximal edge of the ostium of the IA in 54 (34.2%), 73 (46.2%), and 92 (58.2%) of the cases, respectively. Among those with a relative difference <10 and 20%, the diameter of the STJ was larger than that of the proximal edge of the ostium of the IA in 19 and 37 of the cases, respectively.

## Discussion

Our study collective of adequate and high-quality image data of 158 patients is one of the largest studies in Chinese and Asian population screening for anatomic feasibility of endovascular repair for type A aortic dissection.

A potential technique for endovascular repair of TAAD is a valve-carrying endograft. Kreibich et al. (6) conducted an anatomic feasibility analysis on 167 patients and concluded that 66% of the patients were potential candidates for valve-carrying endograft. In this study, moderate to severe aortic regurgitation and cardiac tamponade were not evaluated, and a previous study has suggested that 1/3 of patients with TAAD would present with these two comorbidities (7, 8). Valve-carrying endograft could benefit patients who present with TAAD and moderate to severe aortic regurgitation or cardiac tamponade by completing an endovascular repair for aortic valve and dissected aorta in one stage, but for patients with TAAD, there is still a realistic need for a device specially designed for endovascular repair of the tubular ascending aorta.

Tubular endograft is another option, and only few studies have explored the feasibility of tubular endograft with the proximal landing zone (10/15/20 mm) in the ascending aorta (9–12). The study reported by Fujimura et al. explored the feasibility of an investigational device, the Zenith Ascend (Cook, Bloomington, IN, United States), in Japanese patients. According to their analysis, none of the 131 patients were candidates for Zenith Ascend; most of whom were excluded because of aortic radius <40 mm (mean aortic radius was 31.9 ± 7 mm). Thereafter, Tsilimparis et al. (13) reported the outcomes of endovascular repair with Zenith Ascend for ascending aortic pathologies in Caucasian patients (details of race not mentioned but the procedure was performed in Europe), and there was one death and one case of severe aortic insufficiency related to Zenith Ascend in the perioperative period. Zenith Ascend reduces the proximal landing zone to 10 mm in the tubular ascending aorta starting from the STJ, while 26% of patients were excluded because of insufficient proximal landing zone in a Japanese study. In our series, only 17.7% of the patients had a healthy proximal landing zone for Zenith Ascend. The significant difference in anatomical features, especially in ascending aortic radius of curvature, between Asian and Caucasian patients suggests the importance of setting up a database classified by races for future design and manufacture of ascending endograft devices. A comparison with other literature of anatomical features of the aortic root and ascending aorta of Asian patients with TAAD is shown in Supplementary Table 1.

The concept of extending the endograft into the region between the distal edge of the higher coronary ostium and the STJ makes endovascular repair available for the entire tubular ascending aorta, increasing the number of potential eligible patients. In our series, 39.9% of the patients could provide a landing zone ≥10 mm for fixation. In comparison, only 4.4 and 1.9% of the patients could provide a landing zone ≥15 and 20 mm, respectively. The anatomical characteristic was similar in the Japanese population, where the median distance from the distal coronary artery to the STJ was 6 mm (range 3–11 mm). Wisneski et al. (14) analyzed the anatomic feasibility with the proximal landing zone starting from the higher coronary ostium, but with a different definition of proximal landing zone, which started from the higher coronary ostium to entry tear. In their series, 79.2 and 66% of patients had a proximal landing zone ≥20 and 10 mm, respectively.

**Figure 4:**
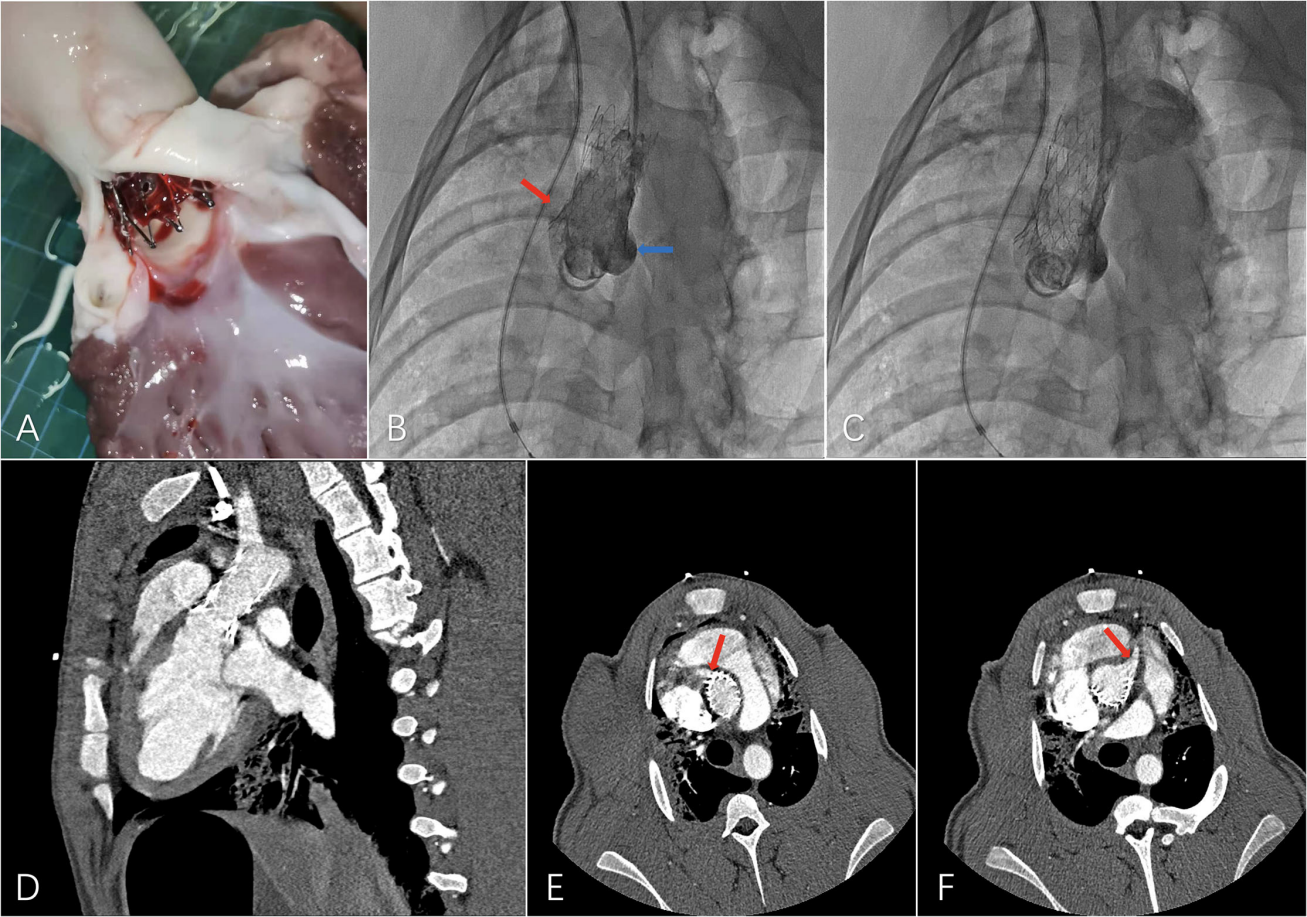
Preliminary experiment with the novel ascending aortic endograft technique on a swine model. **(A)** Dissection after a 1-month period follow-up, **(B)** final angiography showing patent left (blue arrow) and right (red arrow) coronary ostia, **(C)** final angiography showing patent supra-aortic trunks, **(D)** computed tomography angiography (CTA) 1 month after the operation with, **(E)** patent right coronary ostium (red arrow), and **(F)** patent left coronary ostium (red arrow).

A major challenge for extending the proximal landing zone is to maintain sufficient blood flow of coronary ostia on both sides. To avoid ischemia of the myocardium, the bare metal stent part was set between the distal edge of the higher coronary ostium and the STJ, with the junction of the covered part and the bare metal part positioned in the STJ. A preliminary experiment with the ascending aortic endograft was performed on a swine model with healthy aorta. Although the proximal part of the bare metal exceeded the proximal edge of the higher coronary ostium on the greater curvature side, coronary ostia on both sides showed sufficient blood supply in the final angiography after deployment of the ascending aortic endograft. No adverse events such as stent-graft collapse and migration were observed during the 1-month follow-up, and CTA examination after 1 month showed patent coronary ostia on both sides, suggesting the possibility and safety of extending the proximal landing zone into that region. In future preoperative designs, not only for swine models but also for patients, difference in length between the greater and lesser curvature sides should be considered and measured in detail to ensure accurate positioning of the bare metal part in the region between the distal edge of the higher coronary ostium and the STJ to avoid influencing the blood flow of the coronary ostium on each side (Figure 4).

In recent years, a 5% oversizing ratio has been widely accepted in treatment of aortic dissection, but a 0% oversizing ratio has been suggested (15). An oversizing ratio ≥20% could increase the risk of dissection propagation, rupture, and stent-graft-induced new entry tear. A variation rate ≥20% was identified between the diameter of STJ and that of ostium of IA in 66 patients (41.8%). These patients could benefit from tapered tubular ascending endograft.

In cases where the pathology extended distally to the IA, which was 77.2% in our series, revascularization of supra-aortic trunks was necessary to ensure complete exclusion of the pathology and cerebral and upper limb blood supply. The combination of the novel ascending aortic endograft technique with branched aortic arch endograft could be a possible solution. Currently, all branched aortic arch endograft techniques are investigational, including the Dacron Inoue branch stent-graft (16), Zenith Arch endograft (17), Valiant MonaLSA Thoracic Stent Graft System (18), Dual Branch Relay endoprosthesis (19), and thoracic branch endoprosthesis (TBE) (20). Fenestrated aortic arch endograft could be another viable option. In our center, total endovascular aortic arch repair with surgeon-modified fenestrated stent-graft has been performed for treatment of aortic arch pathologies with promising early outcomes (21). Without off-the-shelf products available, the treatment strategy should be decided by the operator after a comprehensive consideration of patient's anatomical characteristics, available devices, and the operator's preference.

Accurate anatomical measurement of the ascending aorta is essential for selecting an appropriate size of the endograft. During the cardiac cycle, the ascending aorta moves in longitudinal and circumferential directions, resulting in pulsatile changes in its diameter. Belvroy et al. (22) found that maximal diameter changes in the ascending aorta could be up to 15.4% in the STJ and 17.8% in the middle of the ascending aorta. Measurements should be performed by electrocardiography (ECG)-gated CT, and operators should consider comprehensively the pulsatile change in diameter and diameter difference in different segments to avoid either too much or too little oversizing ratios. Intra-vascular ultrasound (IVUS) could be a viable option when ECG-gated CT is unavailable to verify the measurements based on CT. In our series, measurements were based on CT, because ECG-gated CT was not routinely performed in our center. However, we highly recommended ECG-gated CT for preoperative measurement, especially for the ascending aorta, in clinical practice.

## Conclusion

Endovascular repair for TAAD has a potential to offer high-risk patients an option for effective life-saving treatment associated with lower rate of morbidity and mortality. Morphological analysis on Asian populations is essential considering the anatomical difference among different races. Our study demonstrates that 39.9% of the TAAD cases would be amenable to endovascular repair with the distance between the distal edge of the higher coronary ostium to the STJ > 10 mm, while including cases where the aortic root is proximally involved by intramural hematoma could increase the availability rate to 56.3%. The study could serve for future device development in Asian populations. Study in healthy and TAAD animal models are the ongoing steps to verify the safety of the new device, after which future clinical studies will be carried out to validate its safety and effectiveness in treatment of TAAD patients.

### Limitation

This was a retrospective study, which was restricted by its sample size and the nature of single-center studies, for patients with TAAD with available high-quality CTA images, and many patients were excluded because of inadequate or unavailable imaging data. Despite taking many measures, systematic errors in measurement could still exist. In this study, no ECG-gated CT was analyzed, which could have significantly influenced the measurements owing to extreme true lumen collapse.

## Data Availability Statement

The raw data supporting the conclusions of this article will be made available by the authors, without undue reservation.

## Ethics Statement

The studies involving human participants were reviewed and approved by Committee on Ethics of Medicine, Navy Medical University. Written informed consent for participation was not required for this study in accordance with the national legislation and the institutional requirements. The animal study was reviewed and approved by Committee on Ethics of Medicine, Navy Medical University.

## Author Contributions

XL, LZhu, LZha, and QL contributed to conception and design of the study. XL and LZhu organized the database. LZha performed the statistical analysis. XL wrote the first draft of the manuscript. CS, SX, HZ, WG, and ZJ wrote sections of the manuscript. All authors contributed to manuscript revision, read, and approved the submitted version.

## Conflict of Interest

The authors declare that the research was conducted in the absence of any commercial or financial relationships that could be construed as a potential conflict of interest.

## Publisher's Note

All claims expressed in this article are solely those of the authors and do not necessarily represent those of their affiliated organizations, or those of the publisher, the editors and the reviewers. Any product that may be evaluated in this article, or claim that may be made by its manufacturer, is not guaranteed or endorsed by the publisher.
