# Supplementary material for: Anatomical Feasibility Study on Novel Ascending Aortic Endograft With More Proximal Landing Zone for Treatment of Type A Aortic Dissection
Source: Front Cardiovasc Med. 2022 Apr 6;9:843551. doi: 10.3389/fcvm.2022.843551 (PMC9019117; doi:10.3389/fcvm.2022.843551)
Supplement: Supplementary file 1 [file Table_1.docx]

Supplementary table 1. Detail of published literature evaluating anatomical features of aortic root and ascending aorta in Asian patients with type A aortic dissection.

| Research | | Liu et al | Fujimura et al^12^ | Zhang et al | Huang et al^11^ |
| --- | --- | --- | --- | --- | --- |
| Distance, mm | Aortic annulus - STJ | — | — | 17.4±4.1 | — |
|  | Higher coronary ostiumSTJ | — | 6 (3-11) | 8.8±2.3 | — |
|  | Higher coronary ostium - primary entry tear | — | 60 (4-250) | — | 36.4±41.0 |
|  | Higher coronary ostium - ostium of IA | 85.6±11.4 | 84 (57-118) | — | 74.4±9.4 |
|  | Aortic annulus - primary entry tear | — | — | — | — |
|  | STJ - primary entry tear | — | 54 (0-244) | 19.1±22.6 | — |
|  | STJ - ostium of IA | — | 78 (52-113) | 69.4±10.7 | — |
| Diameter, mm | Aortic annulus | — | — | — | — |
|  | Sinus of Valsalva | — | — | — | — |
|  | STJ | — | 39 (28-63) | 35.4±2.7 | — |
|  | Ostium of IA | 30.54±6.99 | 41(27-55) | 33.2±3.8 | 31.1±10.0 |
| Radius of curvature, mm | | 59.7±10.27 | 32(10-55) | — | — |

IA, innominate artery; STJ sinotubular junction
